# Hippo-Yap/Taz signalling in zebrafish regeneration

**DOI:** 10.1038/s41536-022-00209-8

**Published:** 2022-01-27

**Authors:** Susanna E. Riley, Yi Feng, Carsten Gram Hansen

**Affiliations:** University of Edinburgh Centre for Inflammation Research, Institute for Regeneration and Repair, Queen’s Medical Research Institute, Edinburgh bioQuarter, 47 Little France Crescent, Edinburgh, EH16 4TJ UK

**Keywords:** Regeneration, Cell signalling, Stem-cell differentiation

## Abstract

The extent of tissue regeneration varies widely between species. Mammals have a limited regenerative capacity whilst lower vertebrates such as the zebrafish (*Danio rerio*), a freshwater teleost, can robustly regenerate a range of tissues, including the spinal cord, heart, and fin. The molecular and cellular basis of this altered response is one of intense investigation. In this review, we summarise the current understanding of the association between zebrafish regeneration and Hippo pathway function, a phosphorylation cascade that regulates cell proliferation, mechanotransduction, stem cell fate, and tumorigenesis, amongst others. We also compare this function to Hippo pathway activity in the regenerative response of other species. We find that the Hippo pathway effectors Yap/Taz facilitate zebrafish regeneration and that this appears to be latent in mammals, suggesting that therapeutically promoting precise and temporal YAP/TAZ signalling in humans may enhance regeneration and hence reduce morbidity.

## Introduction

Many different organisms have the ability to regenerate, although the robustness, efficiency, and scope of this regeneration is varied. Invertebrates such as planarians and Hydra regenerate their entire body such that, when cut in half, each section forms an entire new organism^1–4^. At the other end of the scale, mammalian regeneration is limited, with adult animals often responding to injury with fibrotic scarring rather than regeneration^5,6^. Some mammalian tissues do regenerate, including the skin, intestine, liver, peripheral nervous system, and blood^7–11^, as well as foetal tissues^12^ but this capability is impaired in ageing systems^13,14^, which, along with a general lack of regenerative ability in most tissues, causes high morbidity in humans.

Midway on the scale from complete (invertebrate) to limited (mammalian) regeneration are lower vertebrates, including amphibians and fish. The zebrafish *Danio rerio* has the potential to completely regenerate multiple adult and embryonic organs, including the heart, fin, and many nervous system components^15–20^. First explored in the 1980s by Streisinger^21,22^, the zebrafish is regularly utilised in the study of adult and embryonic regeneration due to their rapid external development, relative low cost, ease of genetic manipulation, scalability, transparent juveniles, and high rate of regeneration, none of which are present in the mouse.

**Fig. 1:**
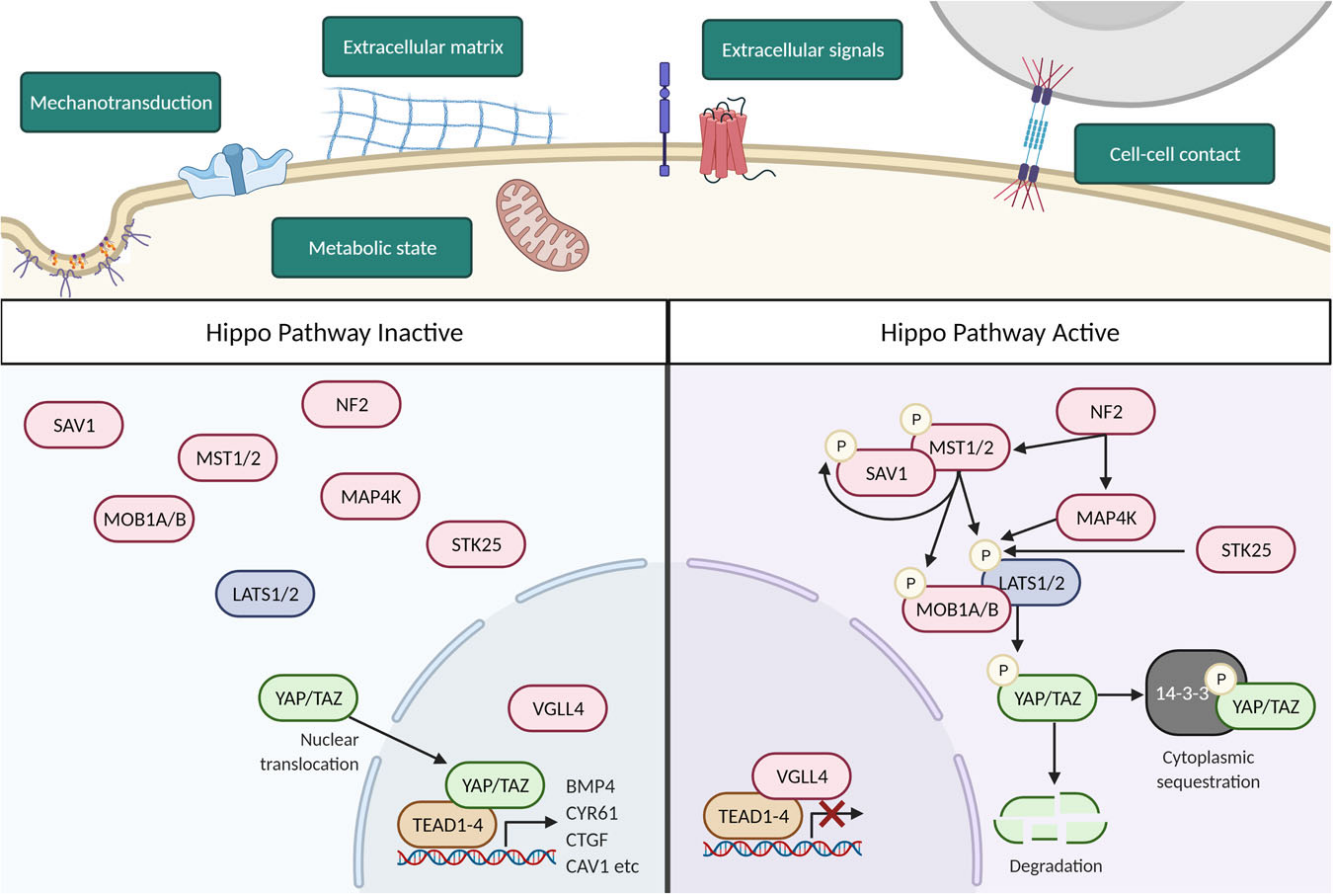
Summary of the Hippo pathway signalling cascade and its stimuli. The Hippo pathway is regulated by the integration of a range of upstream stimuli. This includes mechanotransductive elements (such as caveolae and Piezo signalling), metabolism, extracellular matrix and integrin signalling, transduction of extracellular stimuli via mitogenic growth factor signalling and GPCRs, cell polarity and cell–cell contacts. Activation of the Hippo pathway triggers a phosphorylation cascade that leads to the phosphorylation of the Hippo pathway effectors YAP/TAZ. Phosphorylation of YAP/TAZ redistributes YAP/TAZ to the cytoplasm, blocking TEAD-mediated gene expression. Hippo pathway inactivation prevents YAP/TAZ phosphorylation, allowing their nuclear translocation and hence TEAD-mediated gene expression. Note that MST1/2 (mammalian STE20-like kinase1/2) are encoded by *STK4/3*, and TAZ by *WWTR1*. Figure 1 is created in BioRender.com.

The cellular and molecular drivers of zebrafish regeneration have been the subject of intense research^5,19^. Effective replacement of lost or damaged cells requires a large pool of available healthy cells. Cell pools can be formed by multiple sources, including the activation of resident stem or progenitor cells (differentiation), the reversion of differentiated cells to a more immature pluripotent state (dedifferentiation), or the conversion of one differentiated cell type into another mature cell type (transdifferentiation)^5,23^. On a molecular level, these can be driven by epigenetic and gene expression changes, such as alterations in DNA methylation^5,23–25^, histone modifications^5,26–29^, regeneration-responsive enhancers^28,30–33^, and the activation of a range of key developmental signalling pathways, including Bmp, Fgf, Notch, RA, Shh, and Wnt/β-catenin (summarised in Table 1)^34–77^. In recent years, it has become evident that Hippo signalling (Fig. 1) plays a critical role in developmental and regenerative processes in both zebrafish and mammals. This is associated with the Hippo pathway’s role in regulating cell proliferation and migration, detecting and responding to changes in tissue tension, extracellular matrix, chemical cues, which consequently alter cell fates^78–82^.

**Table 1:** A summary of major non-Hippo signalling pathways involved in zebrafish regeneration.

Signalling pathway	Model	Role of pathway
BMP	Heart^34^	Promotes CM proliferation and dedifferentiation
	Tail Fin^239–241,35,36^	Enhances proliferation and differentiation of osteoblasts in the blastema
Calcineurin	Tail Fin^37^	Regulates regeneration rate for positional information
Fgf	Spinal Cord^188,38^	Increases glial bridge formation, neuronal proliferation, and neurite outgrowth
	Tail Fin^229,39–41^	Promotes blastema formation and regenerative outgrowth Regulates regenerative growth rate
	Lateral Line^42^	Promotes support cell differentiation
Igf	Heart^43^	Enhances CM proliferation
	Tail Fin^44^	Promotes blastema cell proliferation and basal epithelium maintenance
Jak/Stat3	Heart^45^	Promotes CM proliferation
	Lateral Line^46^	Increases progenitor cell proliferation and differentiation
	Liver^47^	Necessary for appropriate timing of progenitor cell-to-hepatocyte differentiation Establishes the correct number of biliary epithelial cells during regeneration
NF-κB	Heart^48^	Promotes CM proliferation and dedifferentiation
Notch	Heart^49^	Enhances CM proliferation
	Spinal Cord^50^	Inhibits motor neuron neurogenesis
	Tail Fin^51,52^	Maintains blastema cells in a proliferative undifferentiated state
	Lateral Line^274,53,54^	Reduces support cell proliferation
	Liver^55–57^	Enhances biliary cell to hepatocyte conversion and differentiation of progenitor cells to biliary epithelial cells
Nrg	Heart^58^	Promotes CM proliferation
RA	Heart^59,60^	Enhances CM proliferation and wound epithelium formation
	Tail Fin^59,61–63^	Increases blastema and basal epidermis formation and patterning during regenerative outgrowth Restricts osteoprogenitor cells to boy ray regions
ROS	Heart^64^	Recruits immune cells and primes heart for regeneration
	Tail Fin^65^	Promotes proliferation of stump epidermal cells
Shh	Heart^43^	Increases CM proliferation
	Spinal Cord^66,67^	Activates motor neuron neurogenesis
	Tail Fin^35^	Promotes proliferation and differentiation of osteoblasts in the blastema
Tgfβ	Tail Fin^41,68^	Enhances cell migration and blastemal proliferation during outgrowth
	Heart^43,69,70^	Promotes CM proliferation and transient scar formation
Wnt/β-catenin	Spinal Cord^185,190^	Increases glial progenitor differentiation into neurons, axonal regrowth, and deposition of pro-regenerative collagen
	Tail Fin^212,239,71–73^	Enhances blastemal cell proliferation and osteoblast dedifferentiation
	Lateral Line^279,53,74^	Promotes support cell dedifferentiation and proliferation, and hair cell formation
	Liver^55,75–77^	Increases differentiation of biliary-derived progenitor cells into hepatocytes

**Fig. 2:**
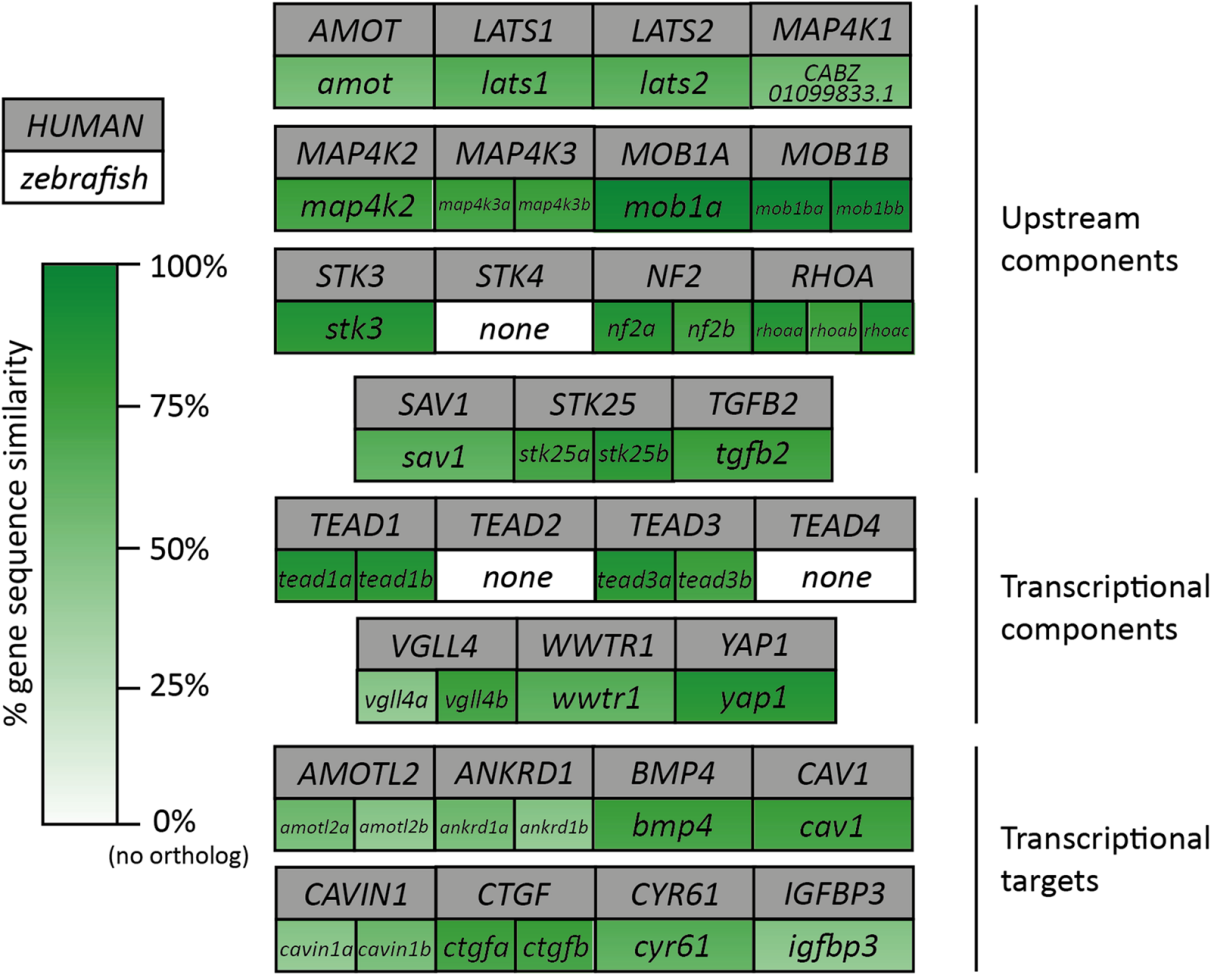
Similarity between selected human and zebrafish Hippo pathway genes. Direct gene sequence comparison between a sample of human and zebrafish Hippo pathway members and transcriptional targets shows a range of similarity scores, emphasizing a high degree of similarities between fish and human genes, while also highlighting that some Hippo pathway components appear to have no direct orthologs present in both species. *WWTR1* encodes TAZ. *STK4* encodes MST1 and *STK3* encodes MST2 (in accordance with the consensus of the Hippo pathway field). *CYR61* is also known as *CCN1* and *CTGF* as *CCN2*. % gene sequence similarity identified using ensembl.org under orthology tab. *ctgfb*, *nf2b*, *map4k2*, and *rhoaa-c* could not be identified as orthologues in this manner, so manual BLAST comparison of genomic sequence (from GRCz11) was performed to give the values indicated.

The core Hippo signalling pathway is comprised of a serine/threonine kinase phosphorylation cascade (Fig. 1), most of which were identified in genetic screens of *Drosophila melanogaster* for tumour suppressor genes^83,84^. Activity of this pathway is regulated by a range of stimuli, including mechanical signalling, cell shape, ECM stiffness, cell polarity, metabolism, and cell:cell contacts^78,79,82,85–90^, which are integrated to stimulate key kinases MST1/2 (the fly Hippo orthologs), STK25, and MAP4Ks when the Hippo pathway is active^87,89,91–93^. These kinases then phosphorylate, and so activate, LATS1/2, which phosphorylate the core Hippo effectors transcriptional co-activator YAP1 and its paralog TAZ on multiple conserved serine residues^86,87,91,92,94,95^. YAP1/TAZ phosphorylation triggers their retention in the cytoplasm via binding to protein 14-3-3, or ubiquitin-mediated degradation^86,87,94–97^. When the Hippo pathway is inactive these phosphorylations do not occur, resulting in YAP1/TAZ nuclear localisation, where they outcompete VGLL4 and bind to transcription factors TEAD1-4^87,98–101^. Binding to TEADs stimulate the expression of a range of pro-proliferative, -oncogenic, -stemness, and -EMT genes, such as *CTGF* and *CYR61*^78,87,90,98–100,102–104^. Additional YAP1/TAZ transcription factors have also been identified^87^, but the most extensively studied are the TEADs. Zebrafish Hippo pathway genes have high genetic orthology to human genes, suggesting that this is an appropriate model in which to study Hippo pathway function (Fig. 2). Here we review the role of the Hippo pathway in the regeneration of a range of organs, including heart, spinal cord, tail fin, lateral line, and liver regeneration, with a focus on the zebrafish.

### Heart regeneration

Cardiovascular diseases are the primary cause of morbidity and mortality globally, with around half of these deaths caused by ischaemic heart disease leading to heart failure^105^. This is due to the limited regeneration capacity of the adult human heart, which responds to heart muscle damage with fibrosis and scarring rather than the reformation of contractile muscle^106^. A similar response is seen in other mammals (such as the mouse), which also show limited cardiac regeneration after experimental injury paradigms^107^. An exception is an enhanced heart regeneration potential in neonatal mice, but this is transient and is lost within the first week of life^108^, coinciding with a decrease in YAP1 transcriptional activity^78^, and the withdrawal of cardiomyocytes (CMs) from the cell cycle^109^. However, this regenerative ability in neonatal mice^108^ highlights that there may be therapeutic potential in reactivating the regenerative capacity in humans.

**Fig. 3:**
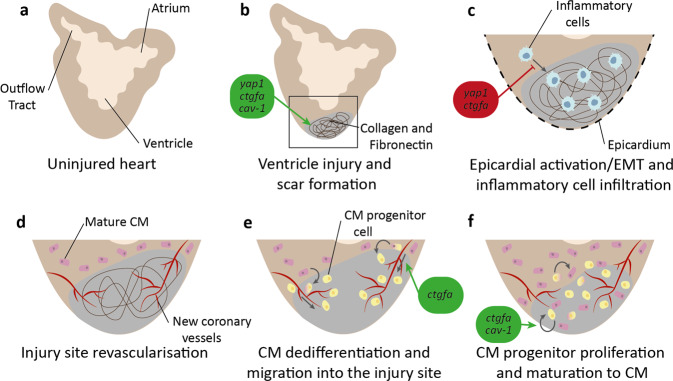
Overview of zebrafish heart regeneration. **a** Structure of the uninjured zebrafish adult heart. **b** Injury at the ventricle apex induces collagen and fibronectin deposition and scar formation. *yap1, ctgfa*, and *cav-1* promote appropriate and transient scar formation. **c** Heart epicardium undergoes EMT and inflammatory cells (blue) infiltrate into the scar. *yap1* and *ctgfa* inhibit inflammatory cell infiltration. **d** New coronary vessels form to revascularize the injury site. **e** Mature cardiomyocytes (CMs) (pink) dedifferentiate into progenitor cells (yellow) and migrate along the new coronary vessels into the injury site. *ctgfa* promotes CM migration. **f** CM progenitors proliferate to create a progenitor cell pool, which matures back to CMs to reform the heart muscle. *ctgfa* and *cav-1* promote cell proliferation.

In contrast to restricted mammalian regeneration, both adult and embryonic zebrafish regenerate their heart fully following injury and even after multiple insults^5,6,15,19,110–113^ (Fig. 3). This extensive heart regeneration is the result of two key characteristics: a high level of existing CM proliferation (around 3% per week, compared to <1% per year in adult mice^114^ and humans^115^), and a permissive extracellular environment that stimulates it^19,116^. One major hurdle and pathological driver in mammalian heart regeneration is the formation of a fibrotic scar and non-permissive ECM at the injury area, replacing dead CMs with non-contractile elements such as collagen or fibroblasts rather than new CMs^106,117^ (Fig. 3B). However, in the zebrafish, although collagen and fibronectin does accumulate and a scar is formed, it is eliminated to allow effective regeneration^113,118–120^. This scarring is regulated by Hippo signalling, with *cav-1*, *yap1*, and *ctgfa* mutants having disrupted scar formation and hence regeneration^121–123^ (see Table 2 for a summary of these phenotypes). Heart injury promotes Ctgfa secretion into the ECM from endocardial cells, where it promotes the expression of pro-regenerative ECM genes (such as fibronectins and collagens)^121^. This expression allows for a transient scar, as shown by *ctgfa* mutants having a larger and more persistent scar, whilst *ctgfa* overexpression speeds scar resolution^121^. Similarly, *yap1* mutants have an altered ECM composition at the injury site, resulting in increased scarring and impaired regeneration at early time points^122^. This alteration of the scar microenvironment by secretion of Hippo pathway transcriptional targets suggests that Hippo signalling may also indirectly regulate the infiltration and proliferation of CMs through a cell non-autonomous mechanism.

**Table 2:** Overview of zebrafish phenotypes seen when Hippo pathway components are disrupted.

Gene	Activity level	Disruption method	Allele created	Model	Phenotype
*amotl2a*	−	MO/TALEN	N/A/*fu45*, *fu46*	LL development^261^	Overproliferation in trailing edge of pLLP Increased pLLP size and cell number Reduced pLLP migration speed Increased number of neuromasts
*cav*-*1α*	−	TALEN	*pd1094*, *pd1104*	Heart regeneration^123^	Impaired recovery after injury, injury-induced CM proliferation, and scar resolution
		MO	N/A	LL development^136^	Reduced number and maturation of hair cells and neuromasts
*ctgfa*	−	TALEN	*bns50*	Heart regeneration^121^	Reduced CM proliferation, expression of pro-regenerative ECM genes, and CM migration along the coronary vasculature to repopulate the wound Increased collagenous scarring
				SC regeneration^197^	Reduced functional recovery after injury, glial cell proliferation and bridging, and axon regeneration
	+	hsp70:*ctgfa* OE plasmid	*pd97*	Heart regeneration^121^	Increased recovery after injury, CM proliferation, resolution of collagen deposition, and expression of pro-regenerative ECM genes
				SC regeneration^197^	Enhanced functional recovery after injury, glial bridging, and axon growth
		CRISPR	*zf3090*	Tail fin regeneration^234^	Increased tissue stiffness, contractility, and ECM deposition
*lats2*	−	CRISPR	*mw87*	Cancer^311^	Increased lethality Formation of peripheral nerve sheath tumours by 3mpf
*nf2a*	−	MO	N/A	Liver development^299^	Hepatomegaly, dilated bile duct, and extrahepatic choledochal cysts
*sav1*	−	CRISPR	*mw95*	Liver development^301^	Biliary dysgenesis, altered hepatocyte morphology and polarity, and biliary cell dysplastic morphology and increased expansion
*stk3*	−	TALEN	*mw96*	Liver development^301^	Biliary dysgenesis, altered hepatocyte morphology and polarity, and biliary cell dysplastic morphology and increased expansion
*wwtr1*	−	MO	N/A	Tail fin regeneration^149^	Lack of skeletal ossification
*yap1*	−	TALEN	*mw48*	Heart regeneration^122^	Improper scar formation Reduced ability to secrete collagen at the injury site Increased macrophage infiltration in the scar, monocyte chemotactic gene expression, space between the epicardium and myocardium, and CM proliferation
				LL regeneration^277^	Reduced progenitor cell maturation and proliferation
				Liver development^147^	Reduced liver size
		hsp70:DN-Yap plasmid	*zf621*	SC regeneration^193^	Impaired functional recovery after injury, axon growth, and glial bridging
				Tail fin regeneration^232,233^	Reduced recovery after injury, cell proliferation, and osteoprogenitor differentiation into osteoblasts Defects in bone formation
		5 µM verteporfin	N/A	LL regeneration^277^	Defective supporting cell, hair cell and mantle cell proliferation and hair cell maturation
				LL development^259–262^	Reduced number of neuromasts and hair cells, pLLP size, number of cells in the pLLP, mechanoreceptor differentiation, and Wnt signalling component expression
	+	Tol2 (myl7:3SA-myc *yap1*)	N/A	Heart regeneration^122^	Increased CM proliferation
		hsp70:CA-Yap plasmid	*zf622*	Tail fin regeneration^232^	Impaired recovery after injury, increased cell proliferation
		CA-Yap1 mRNA injection (in *lpar2b* MO)	N/A	LL development^262^	Increased pLLP size, and number of neuromasts and proliferating cells in the pLLP
		I-SceI (*lf:Yap1*)	N/A	Liver development^300^	Hepatomegaly
*yap1*;*wwtr1*	−	CRISPR	N/A	SC regeneration^193^	Impaired functional recovery after injury

This table is non-exhaustive and primarily covers developmental and regenerative phenotypes described in this review. Many other Hippo pathway mutants and morphants exist (e.g. *wwtr1* alleles *bns35*, *swu46*, *swu47*, *va4*, *mw49*, *ncv114*, and *fu55*). See individual gene pages on zfin.org for a complete list.

The immune system creates a permissive microenvironment for regeneration (Fig. 3C). This is clearly demonstrated in the medaka, a teleost species closely related to the zebrafish. The medaka displays limited heart regeneration, a finding that is surprising considering their evolutionary similarity to the zebrafish^124,125^. This limited regeneration is, at least in part, due to the medaka’s delay in macrophage recruitment to the injury site^124^. When recapitulated in the zebrafish by clodronate liposome-mediated macrophage depletion, these macrophage defects cause compromised neovascularisation and CM proliferation and consequently severe defects in heart regeneration^124^. This is due to the role of macrophages and other immune cell components such as T_reg_ cells in many areas of cardiac regeneration, including enhancing neovascularisation, CM proliferation, and scar resolution via the production of pro-regenerative factors, with inhibition of inflammation and timely immune cell recruitment inhibiting regeneration^124,126–129^.

However, this pro-regenerative effect of the immune system is not simple. Yap1-Ctgfa signalling, shown to enhance cardiac regeneration, also negatively regulates the migration and infiltration of macrophages into the injury site^121,122^, suggesting that inhibiting macrophage infiltration promotes cardiac regeneration. Similarly, *yap1* KO fish have increased macrophage infiltration in the scar and increased monocyte chemotactic gene expression^122^, and *ctgfa* KO promotes the chemokine receptor gene *cxcr3.1* in the heart to increase M1 macrophage polarisation and so enhance inflammatory signalling^121^, and both KO lines have defective regeneration. This apparent contradiction may be due to differences between experimental paradigms in investigating immune cell function in regeneration—it has been shown that the type of immune cells recruited, and the different regenerative stages alter the functional role of the immune system in regeneration^128^. An alternative explanation for this apparent discrepancy could be due to the requirement for tight spatio-temporal control of the immune system function during regeneration. This is shown by disruption of reparative regeneration after both immune system hyperactivation^121,122^ and excessive inhibition^124,126,127,129^. Another potential reason for the inconsistency is that various immune cell types likely react differently to the injury, and so the Hippo pathway may respond in a range of ways to the same trigger. Therefore, the extent of activation or inhibition in these studies will greatly impact the results. Further in-depth studies are needed in order to fully elucidate the detailed spatiotemporal inflammatory response including revealing the exact immune cell types involved in regeneration and thereby the role of the Hippo pathway in the immune system’s contribution to cardiac regeneration.

Hippo pathway signalling has also been linked to the epicardium, which is activated after heart injury in the zebrafish (Fig. 3C)^15,130^. The epicardium promotes regeneration, potentially by functioning as a cellular scaffold that generates epicardial-derived cells which differentiate into myofibroblasts and perivascular fibroblasts in the injured myocardium^131^. This may then act in a paracrine manner to induce CM proliferation and neoangiogenesis^131^. Epicardial activation has not yet been linked to the Hippo pathway in zebrafish heart regeneration. However, in the developing mouse, Hippo components are expressed in both the proepicardium and epicardium, and deletion of either Yap or Taz in the mouse gives coronary defects and impacts on epicardial cell proliferation, EMT, and specification of cell fate^132^. Similar developmental cardiac defects can be seen in a range of Hippo pathway component mutants in the zebrafish^112,133–152^, suggesting that this role of the Hippo pathway may be conserved between mammals and teleosts.

After injury, existing differentiated CMs undergo limited dedifferentiation, upregulate the embryonic cardiogenesis gene *gata4*, and proliferate^113,153–156^. These CMs migrate to the injury site along newly-formed coronary vasculature^157–162^ (Fig. 3E), where they proliferate further and differentiate to replace dead CMs and form new functional heart muscle^163^ (Fig. 3F). CM proliferation is promoted by a range of signalling pathways, including Nrg, Tgfβ, Igf, and the Hippo pathway (Table 1). Disruption of the Hippo pathway-regulated genes *cav-1α* and *ctgfa* inhibits CM proliferation and repopulation of the injury area^121,123^. Similarly, TGFβ-mediated activation of regulatory elements upstream of *ctgfa* promotes CM proliferation at the injury site^32^. *cav-1α* and *ctgfa* are induced after injury in epicardial and endocardial cells respectively, suggesting a role for these cells in cell non-autonomous regulation of CM function, such as in ECM secretion in response to extracellular stimuli^121,123^. Disruption of *cav-1α* and *ctgfa* results in defective heart regeneration^121,123^, whilst overexpression of *ctgfa* and *yap1* has the opposite effect^121,122^. Disrupting Hippo signalling in mammals gives comparable results. In pigs, CM-specific knockdown of *Sav* (which results in increased YAP activity^164,165^) increases CM proliferation and improves heart function after myocardial infarction^165^. Similar outcomes are observed when *Yap1* is disrupted in mice, causing heart regeneration defects through decreased CM proliferation^166–171^, whilst heart regeneration (and CM proliferation) is stimulated after *Yap1* activation^167–169^, potentially due to the Hippo pathway’s link to cytoskeletal and ECM regulation^170^. However, the opposite effect is observed when Hippo signalling is disrupted in murine cardiac fibroblasts^172,173^. Deletion of *Yap1*/*Taz* in these fibroblasts results in improved cardiac function after myocardial infarction through modulation of the fibrotic and fibroinflammatory response^172^. Enhanced Yap1/Taz signalling (through either Yap1 overexpression or *Lats1*/*2* deletion) has the opposing effect, with mice displaying elevated fibrotic responses^173,172^. This apparent contradiction between the role of the Hippo pathway in CMs and cardiac fibroblasts supports a model where the Hippo pathway functions differently in different cell types.

CMs in zebrafish *ctgfa* mutants also fail to migrate along the coronary vasculature to infiltrate the wound, despite no changes in revascularisation, potentially as a result of alterations in cytoskeletal gene expression in a cell autonomous regulation of CM infiltration^121,123^. Supporting this, data using in vitro primary rat cultures of cardiac fibroblasts show that *Yap1* siRNA-mediated knockdown reduces expression of factors associated with cytoskeletal motility and ECM adhesion, although these results have not been recapitulated in zebrafish, CMs, or in vivo^122^.

In summary, the Hippo signalling pathway enhances cardiac regeneration by temporal activation of Yap1/Taz and promotes normal cardiovascular development. Yap1/Taz promote appropriate scar formation and potentially prevent overactivation of the immune response, which, when combined, increase scar resolution, spatiotemporal CM proliferation, and thereby cardiac regeneration. Taking advantage of this regenerative capacity may hold therapeutic potential in the treatment of human MI. For example, pharmacological regulation of the Hippo pathway could modulate CM proliferation and fate plasticity^156,174^, promoting scarless healing in the adult heart and reducing disease burden. Recent work disrupting Hippo signalling in pigs after myocardial infarction^165^ suggests, in a clinically relevant model system, that this could be possible. However, precise cell type-specific modulation of the Hippo pathway will be vital to realise its full potential, as the Hippo pathway has been shown to have different functions in the cell types involved. For example, heart function is improved after injury in mammals when YAP activity is increased in CMs^165^ but also when *Yap1/Taz* is deleted in cardiac fibroblasts^172^.

### Spinal cord regeneration

**Fig. 4:**
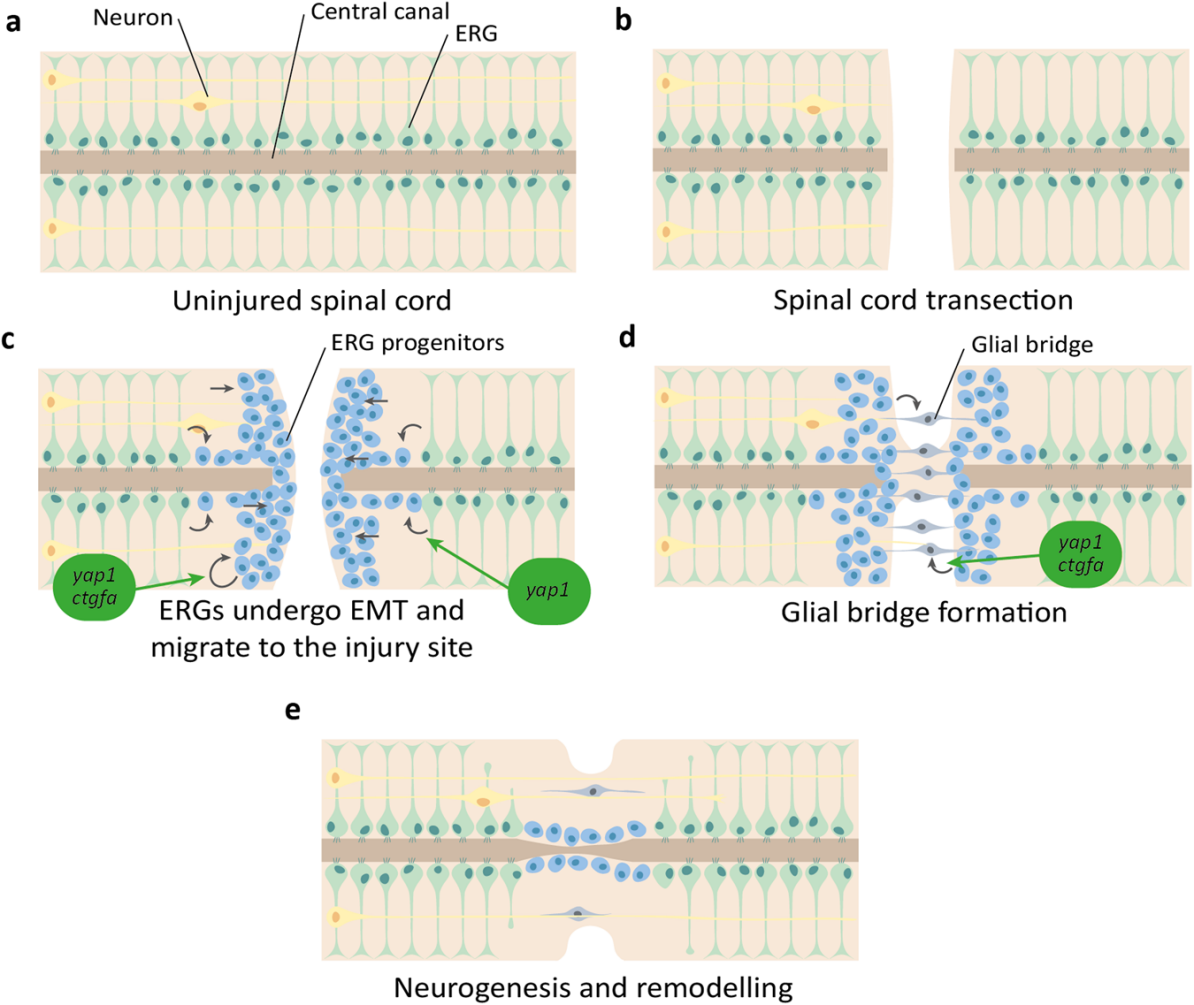
Overview of zebrafish spinal cord regeneration. **a** Structure of the uninjured spinal cord, with ependymal radial glia (ERG) (green) lining the central canal and motor neurons (yellow). **b** Spinal cord transection disrupts neuronal processes. **c** ERGs undergo EMT to form ERG progenitors (blue) and migrate to the site of injury. *yap1* promotes EMT of ERGs, and *yap1* and *ctgfa* promote progenitor proliferation. **d** ERG progenitors extend processes across the injury site to form a glial bridge (grey). *yap1* and *ctgfa* promote the formation of the glial bridge. **e** Neuronal processes extend across the injury site, guided by the glial bridge to promote remodelling and reformation of the spinal cord.

The Hippo pathway is also associated with regeneration after spinal cord injury (SCI) in the zebrafish. After SCI in humans and other mammals, the affected axons and neurons are destroyed and a non-permissive scar is formed in the place of new cells, commonly resulting in lifelong disability^175–177^. However, both adult and larval zebrafish robustly and effectively regenerate their spinal cords after injury, with viable axon regrowth over the lesion site and return of full swimming function within weeks after injury^18,19,178–182^ (Fig. 4).

For functional recovery in the spinal cord, new and existing cells must proliferate, migrate to the injury site, bridge the lesion, and differentiate to reintegrate with existing distal neuronal circuitry^183^. Neurogenesis from tissue-resident progenitors is a vital step for this to occur in zebrafish, which is promoted by multiple signalling pathways, including Wnt/β-catenin, Fgf, Shh, and is inhibited by Notch signalling (Table 1). The tissue-resident progenitors responsible for cell proliferation and bridging are thought to be the ventral ependymal radial glia (ERG)^181,184–186^. These cells have general functions during development and adulthood in maintaining spinal cord homoeostasis such as sealing the blood-brain barrier and maintaining ion balance, but also proliferate and differentiate into a range of neuronal cell types after injury^181,183,187^.

To allow new cell processes to traverse the lesion site, a glial bridge is formed. After injury, ERGs migrate to the lesion and elongate to form an astroglial bridge over the lesion, along which axons can grow to innervate distal targets (Fig. 4C–E). This is driven by pro-regenerative gene expression (e.g. *col12a1a/b* and *tenascin-c*), interactions with other cell types such as Schwann cells, and additional environmental cues^180,182,184,188–190^. Zebrafish glial bridging shares clear morphological and functional similarities with the bridging observed during mammalian peripheral nerve regeneration (which occurs to a much greater extent than mammalian CNS regeneration)^183,191–193^, indicating that this common process may be manipulated in the human for therapeutic benefit.

In order to induce glial cells to undergo bridging, ventral ERGs undergo an epithelial-to-mesenchymal transition (EMT)^193^ (Fig. 4C). EMT is a common feature of many cells activated by injury, and is linked to stem cell activation, increased cellular plasticity, and tissue remodelling^194–196^. Glial EMT is both necessary and sufficient to induce glial bridging, and is linked to Yap1-Ctgfa signalling^193,197^. *yap1*, *wwtr1* (gene encoding Taz), and *ctgfa* are upregulated following SCI, with *yap1* and *ctgfa* expression localised to bridging glia and ventral ERGs^193,197^. As well as inducing *ctgfa* expression in ventral ERGs, Yap1 promotes *twist1a* expression^193^. *twist1a* is an established EMT marker, activation of which directs a mesenchymal transition in Ctgfa^+^ ERGs, promoting glial bridging and functional spinal cord repair^193^.

Similar to heart regeneration, one major difference between the zebrafish and mammalian response to SCI is the formation of a glial scar. SCI causes vascular damage, oedema, and inflammation, resulting in widespread gliosis, necrosis, and apoptosis that eventually forms a glial/fibrotic scar in mammals, stretching beyond the site of the initial trauma and acting to prevent secondary damage but also preventing axon regrowth^176,198,199^. There is no significant scarring in the zebrafish, so there is no experimental work linking the Hippo pathway in zebrafish to scar resolution, however siRNA-mediated knockdown of the YAP1/TAZ-TEAD target gene *Ctgf* in rats reduces the glial scar and hence improves regeneration after SCI^200^, suggesting that YAP1/TAZ signalling may promote scar formation or impair scar resolution and outlining a potential therapeutic target for SCI treatment in mammals.

Loss of function mutations of *yap1*, *wwtr1*, and *ctgfa* all result in impaired functional recovery after SCI, with *ctgfa* and *yap1* disruption causing a glia-specific cell proliferation reduction, resulting in impaired bridging and axon regeneration across the lesion site^193,197^. Exogenous administration of human CTGF to these *ctgfa* mutants reversed this defect^197^. This finding, and the similar finding that heart scar formation is larger and more persistent in *ctgfa, yap1*, and *cav-1* mutants^121–123^ appears in contrast to that seen in the rat glial scar^200^, which found that knockdown of CTGF increased recovery through the clearance of scarring, and the current clinical trials which are targeting CTGF to reduce fibrosis and scarring^201^. This may be due to species differences in the function of the Hippo pathway, but this is not supported by the relatively high translatability of other studies between rodent and zebrafish. An alternative explanation might be that Yap/Taz-Ctgfa signalling has opposing effects at different stages of spinal cord regeneration, or that strictly regulated temporal activation/repression of signalling is key, although studies of this in mammals must be performed after the scar has been resolved, which currently presents an experimental challenge.

Yap1 signalling is also associated with the regenerative role of glial cells in other parts of the CNS, such as the retina. In the zebrafish, retinal damage induces reprogramming events where Müller glia are converted to a highly proliferative progenitor-like state, dividing asymmetrically to replace lost photoreceptors^202–207^. *Yap1* knockdown blocks Müller glial cell proliferation and neurogenesis after light damage of the zebrafish retina^208^, suggesting a common role for *yap1* in the regenerative functions of glial cells. Mammalian retinas usually do not have a proliferative, pro-regenerative, Müller glia response to injury. However, in the mouse, YAP promotes glial reprogramming, with YAP activation inducing Müller glia reprogramming to a highly proliferative, progenitor-like cell^202,204^. This suggests that promoting Yap1 signalling therapeutically may also promote CNS regeneration in humans.

These findings propose a model in which Yap1 senses the mechanical stress caused by SCI, enhancing *ctgfa* and *twist1a* expression to activate a pro-EMT and pro-proliferative transcriptional programme in ventral ERGs, promoting glial bridging, axon regeneration, and, consequently, functional recovery^193^. This model suggests that enhancing scar resolution, promoting EMT, enhancing CTGF signalling at later stages of regeneration, and identifying CTGF-responsive spinal cord cells may allow for the identification of a therapeutic target to promote mammalian spinal cord regeneration^197^. Targeting CTGF has been investigated in a variety of preclinical and clinical trials for multiple conditions, including muscular dystrophy and pancreatic cancer. For example, the monoclonal antibody Pamrevlumab has shown promise in trials for idiopathic pulmonary fibrosis^209,210^. However, these trials involve the inhibition of CTGF activity, rather than the enhancement that may be required to promote recovery^201,211^. Consequently, further insights must be obtained before translating these findings into an effective treatment option in humans.

### Tail fin regeneration

**Fig. 5:**
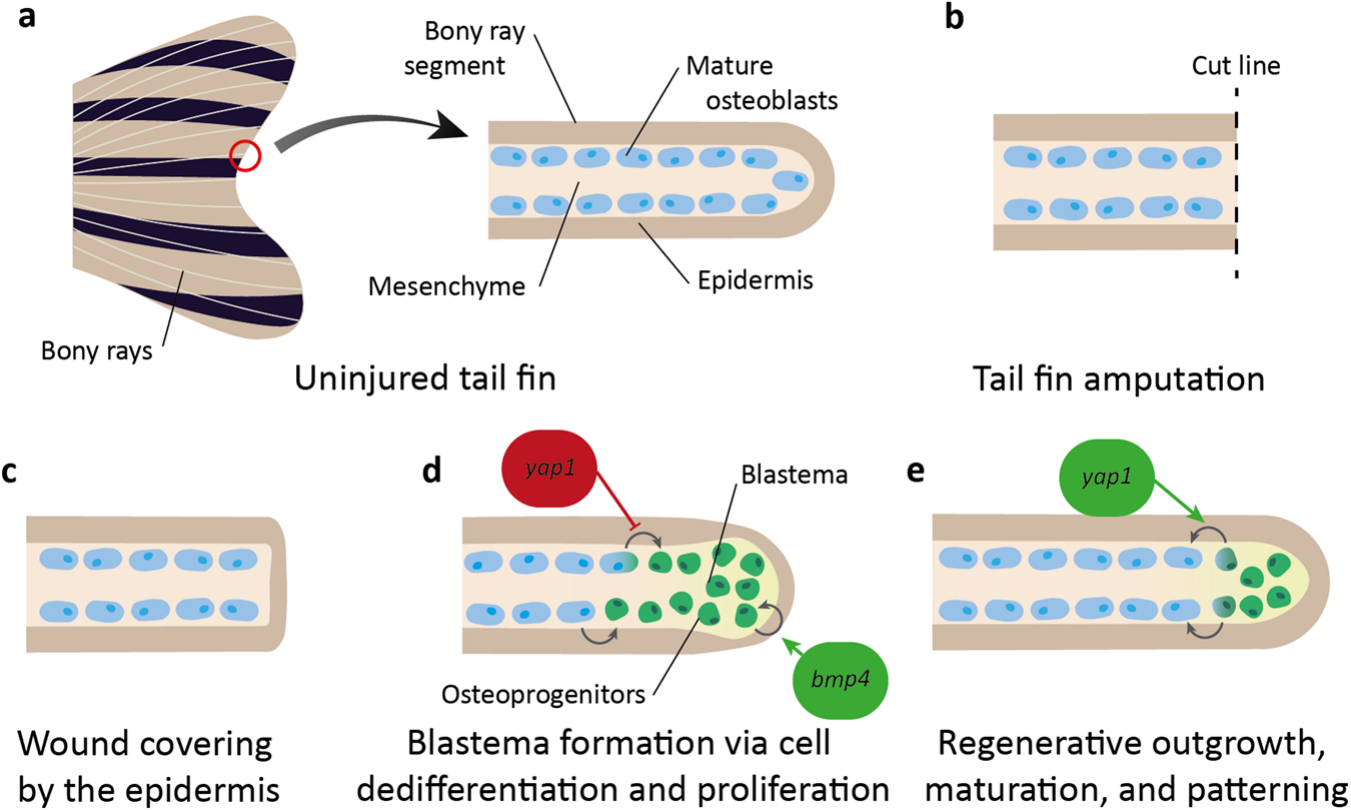
Overview of zebrafish tail fin regeneration (adult), focussing on osteoblast regeneration of bony rays. **a** The uninjured tail fin of the adult zebrafish is formed of many bony rays, which each consist of epidermis surrounding mature osteoblasts (purple) in the mesenchyme. **b** Amputation of the tail fin disrupts the bony ray segment. **c** In the initial stages of tail fin regeneration the epidermis covers the wound. **d** Osteoblasts and other mature cells dedifferentiate and proliferate at the wound tip to form a blastema with osteoprogenitors (green). *yap1* inhibits osteoblast dedifferentiation and *bmp4* enhances blastema cell proliferation. **e** The bony ray segment extends through maturation of the progenitor cells back to their original cell type. *yap1* promotes osteoprogenitor maturation.

Zebrafish and other teleosts regenerate their fins completely after multiple consecutive amputations^212^, a phenomenon that was studied as early as the 18^th^ century^213^, and by the regeneration pioneer T. H. Morgan at the turn of the 20^th^ century^214–216^. Fin regeneration occurs through epimorphic regeneration, a process characterised by the presence of a blastema early in regeneration (Fig. 5). This mass of undifferentiated proliferating progenitor cells at the site of injury is formed by mature cell dedifferentiation, which can then differentiate back into mature cells to generate an actively growing tissue that replaces the lost appendage^217^.

There is not yet direct evidence for a role for the Hippo pathway in dedifferentiation in the zebrafish caudal fin blastema, but in other in vivo models, both mammalian and invertebrate, the Hippo pathway maintain stemness, promote proliferation, and revert differentiated cells to a progenitor cell state^81,218–224^. In the zebrafish, dedifferentiated cells proliferate to form a large pool of progenitor cells in the blastema (Fig. 5D).

Blastema formation is enhanced and maintained by a range of developmental signalling pathways, including Hippo, Wnt/β-catenin, Igf, Notch, Fgf, Shh, Tgfβ, (Table 1) as well as inflammatory signals such as Il1β and Hsp90α^225–227^. The concentration gradient of these signalling pathways gives positional information along the proximodistal axis of the injured tissue, ensuring that structures are reformed at the correct location and that the tissue grows at an appropriate rate, halting when the previous size and shape is reached^16,228–231^. Hippo signalling is one such signalling pathway with activity changes in proximodistal expression. In the high cell density distal blastema, Yap1 is mainly cytoplasmic (and so inactive), whilst in the low density proximal blastema, it becomes mainly nuclear (active)^232^. Yap1 is also localised to α-catenin and F-actin when in the cytoplasm^232^. This suggests that the heterogeneous cell densities within the blastema could be transduced through cell junctions and the cytoskeleton^232^. These mechanical properties then impact Yap1 localisation, which alters the regenerative capacity of the fin^232^. For example, *yap1* disruption impairs cell proliferation and alters key signalling pathways, including promoting Wnt and reducing Bmp signalling after fin injury^232,233^. This results in an accumulation of osteoprogenitors and prevention of osteoblast differentiation, and so defective regeneration^233^. Ctgfa levels are also increased following fin injury, and disruption of its regulatory sequences induces increased tissue stiffness and ECM deposition^234^.

Tail fin progenitor cells are not multipotent. Instead, cells remain lineage restricted^235,236^. The osteoblast is one such cell type. After injury, these cells dedifferentiate, proliferate, and mature to only give rise to osteoblasts in the regenerate (Fig. 5)^236–238^. More specifically, injury induces differentiated mature osteoblasts close to the injury site, which usually form the bony rays of the fin, to lose expression of late and intermediate osteoblast differentiation markers (such as *osteocalcin* and *osterix*) and undergo a Wnt/β-catenin-mediated EMT to gain progenitor markers and generate osteoprogenitor cells, which migrate to the blastema and proliferate in a Fgf-dependent manner^237,239^. These progenitors then undergo Bmp-mediated maturation into osteoblasts^239^ (Fig. 5D), a process that is associated with the Hippo pathway^233,239^. This link to osteoblast formation and function is most dramatically illustrated by *wwtr1* disruption in embryonic zebrafish, which results in a complete lack of skeletal ossification^149^. Similarly, disruption of *yap1* results in major bone defects and impaired fin regeneration, caused by an inhibition of osteoprogenitor cell maturation, giving an increased osteoprogenitor pool with a downregulation of intermediate and mature gene markers^233^. These defects are mediated by a reduction in Bmp signalling (which usually promotes maturation into osteoblasts^239^). In wild-type fish, Yap1 promotes Bmp signalling in a cell non-autonomous manner, restricting osteoprogenitors to the distal blastema (where Yap1 is inactive), and promotes osteoblast formation in the proximal blastema (where Yap1 is active)^233^. *bmp4* is also associated with tail fin regeneration. Bmp4 is expressed in the distal blastema, and its inhibition reduces fin outgrowth after injury due to reduced proliferation of blastema cells^240,241^. This data suggests that Yap1 functions in the blastema to mechanotransduce tension changes and control the fate and migration of specific cell types in the amputated fin, regulating the precise control of tissue growth, potentially through the expression of ECM factors such as Ctgfa^232,234^.

The Hippo pathway is also associated with the differentiation of osteoblasts from mesenchymal stem cells (MSCs) during development, which generate neurons, adipocytes, skeletal muscle, and osteoblasts^242^. In in vitro studies, TAZ promotes osteoblast differentiation from MSCs via activation of Runx2-dependent gene transcription whilst inhibiting adipocyte differentiation via repression of PPARγ signalling^149^. CTGF also promotes osteoblast differentiation from MSCs in vitro^243^. Similar data are observed in mice, where YAP1 and TAZ promote bone formation and repair through their regulation of the osteoblast lineage^244,245^. Osteoblast lineage-specific *Yap1* KO mice have reduced osteoblast differentiation and increased adipocyte formation, an effect that is diminished following increased β-catenin expression, demonstrating the importance of Wnt/β-catenin signalling in this process^245^. However, the role of the Hippo pathway in osteoblast differentiation is contested, with some in vitro studies suggesting that YAP1/TAZ suppress osteoblast differentiation and bone formation, and increase adipogenesis^246,247^, so more work is required to elucidate this complexity.

Zebrafish tail fin regeneration is most closely associated with limb regeneration, which does not occur in mammals or other higher vertebrates, although the mouse has been found in some instances to regenerate the digit tip in both newborns and adults^248^. Appendage regeneration does occur in certain amphibians such as salamanders as well as some invertebrates, and the *Drosophila yap1* ortholog *yki* has been shown to promote wing disc regeneration^249^. Regeneration of an entire limb in mammals appears unlikely, but work in the zebrafish tail fin and other systems suggests that Hippo signalling may play an important role and promoting it could enhance regenerative capacity of specific aspects of limb regeneration, such as enhanced bone regeneration after breaks.

### Hair cell regeneration in the lateral line

**Fig. 6:**
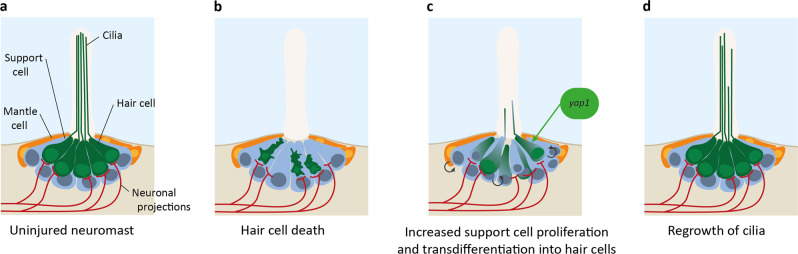
Overview of neuromast regeneration. **a** Uninjured neuromasts consist of hair cells (green) with cilia projecting into the external liquid, support cells (blue), mantle cells (orange), and afferent sensory neurons (red) that project to the brain. **b** Administration of aminoglycosides or Cu^2+^ causes specific hair cell death. **c** Support cell proliferation increases and cells transdifferentiate into hair cells. *yap1* promotes support cell transdifferentiation. **d** Hair cell cilia regrowth restores neuromast function.

The lateral line is a mechanosensitive organ in fish and other aquatic amphibians that detects motion of the external liquid, aiding feeding and social behaviour as well as orientation in currents. In zebrafish, this rapidly developing organ is formed of sixty small clusters of cells (termed neuromasts) in adulthood (expanded from an initial eight in larvae)^250^, located along with the head (anterior lateral line) and trunk (posterior lateral line, pLL) in stereotyped positions^17^. Neuromasts consist of a group of hair cells with stereocilia projecting out of the skin and into the surrounding water, mechanical movement of which triggers sensation, and surrounding interdigitating supporting cells and mantle cells (Fig. 6A). Hair cells are innervated by ribbon synapses with afferent sensory neurons^17^ that project to the hindbrain, where they exhibit a somatotopy similar to the tonotopy seen in mammalian cochlear afferent projections^251^.

During early zebrafish development, a pLL primordium (pLLP) is generated behind the otic vesicle, forming a mass of cells that migrates along the flank beneath the skin, depositing protoneuromasts at periodic intervals^252–255^. The deposition of protoneuromasts and their development into mature neuromasts is mediated by Wnt/β-catenin, Notch, and Fgf signalling pathways, and is reviewed elsewhere^17,256^. These migrating cells must maintain a cohesive structure through high levels of expression of E-cadherin and tight junctions. In mammalian epithelial cells, E-Cadherin is a key upstream regulator of YAP1/TAZ^257,258^, indicating a potential role for Hippo signalling in this process. In fact, the Hippo pathway is linked to lateral line development in the zebrafish, as indicated by the induced expression of Yap1, Amotl2a, and Cav-1α in the developing lateral line^136,259–261^, and how disrupting these proteins functions impact lateral line formation. Downregulation of Cav-1α reduces the number of neuromasts formed^136^ and disruption of *yap1* triggers a range of phenotypes, including a reduction in primordium size, reduced number of neuromasts, and a decrease in hair cell number^259–262^. Amotl2a negatively regulates Yap1 in the developing lateral line, limiting proliferation and so restricting the size of the pLLP, coupling with Notch signalling (which upregulates Yap1 to promote proliferation) to ensure correct pLLP size is reached^259,261^.

Analysis of the transcriptome of *yap1*-deficient embryos shows multiple gene expression changes, including those involved in the Wnt/β-catenin signalling pathway^260^, and lysophosphatidic acid (LPA)^262^. One of these factors is Prox1a, a target of β-catenin that aids hair cell differentiation in the lateral line^260,263^. Analysis of *yap1-* and *prox1a*-deficient embryos shows that *yap1* deficiency recapitulates the *prox1a* deficiency phenotype of reduced hair cell number and impaired mechanoreceptor differentiation. These *yap1* phenotypes are rescued by the administration of *prox1a* mRNA, suggesting that Yap1 functions by promoting Prox1a activity, so regulating hair cell maturation^260^. In the lateral line, the LPA receptor Lpar2b is expressed in the pLL and neuromasts, and its loss-of-function phenocopies *yap1* KD^262^. LPA inhibits the Hippo kinase module, consequently activating YAP1/TAZ^264,265^ and stimulating cell proliferation, migration, and differentiation^262,266^. In the zebrafish specifically, LPA affects early development, promoting vascular and midline development, left-right patterning, and cell migration during gastrulation, amongst others^267–270^. In the pLL Lpar2b regulates Yap1 phosphorylation, suggesting that LPA signalling controls both primordium size and neuromast number by regulating Yap1 activity^262^. These results suggest that the Hippo pathway promotes appropriate size and cell function in lateral line development through a range of signalling pathways, many of which are also associated with other developmental processes.

The high regenerative capacity of the amphibian lateral line was first observed in the salamander^271,272^ but has since been observed in multiple organisms, including the zebrafish^273^ (Fig. 6). This is in contrast to the limited regeneration of mammalian hair cells, e.g. of the inner ear^256^. The majority of regenerated lateral line hair cells are formed by symmetric asynchronous division of support cells in the first 20 hours post injury^254,274,275^, where mitotic division of one support cell gives rise to two hair cells^276^ (Fig. 6C). The molecular and cellular triggers of this regeneration include pathways involved in lateral line development—Wnt/β-catenin, Notch, and Fgf signalling—as well as novel factors such as the Jak/Stat3 pathway (Table 1), which balance self-renewal, hair cell differentiation, and the risk of overgrowth.

The Hippo pathway links to lateral line regeneration^277^. The expression pattern of supporting cells during regeneration is reminiscent of expression in the migrating primordium during lateral line development, which is silenced when leading progenitors differentiate into mature supporting cells and hair cells^259,278,279^. This includes the expression of Hippo components *cav-1* and *ctgfa*, which are upregulated in the support cells of both the zebrafish lateral line and the mouse inner ear^280^. In addition, after severe hair cell injury, Yap1 is activated in hair cell precursors, and regeneration is impaired in *yap1* mutants^277^. Yap1 activation may occur through cell junction damage and resulting loss of junction-associated proteins such as Amotl2a, which usually restricts Yap1 activity in the lateral line^261,277^. Activated Yap1 upregulates *lin28a* transcription, an RNA-binding protein that regulates the translation of mRNAs involved in developmental timing, pluripotency and metabolism^281^. This promotes a Yap1-lin28a-let7-Wnt signalling axis that is both necessary and sufficient to promote progenitor cell activation and hence neuromast regeneration. The Yap1-lin28a-let7-Wnt signalling axis has other roles in dedifferentiation, including zebrafish retinal regeneration, mammalian embryonic inner ear development, and in vitro reprogramming of stem cell cultures^282–285^.

In summary, Yap1/Taz signalling in progenitor support cells is triggered after hair cell injury, promoting their differentiation towards hair cells via a Wnt signalling pathway, and enhancing recovery. Promoting Yap1/Taz signalling may also have therapeutic benefits in humans. The hair cells of the inner ear do not regenerate^256^ but have high similarity to zebrafish lateral line hair cells. This includes similar expression patterns of mechanosensitive ion channel and tip link genes and responses to key signalling pathways and ototoxic insults^256,286–290^, and so targeting the Hippo pathway to promote the regeneration of inner ear hair cells to combat age-related hearing decline may be a viable approach.

### Liver regeneration

**Fig. 7:**
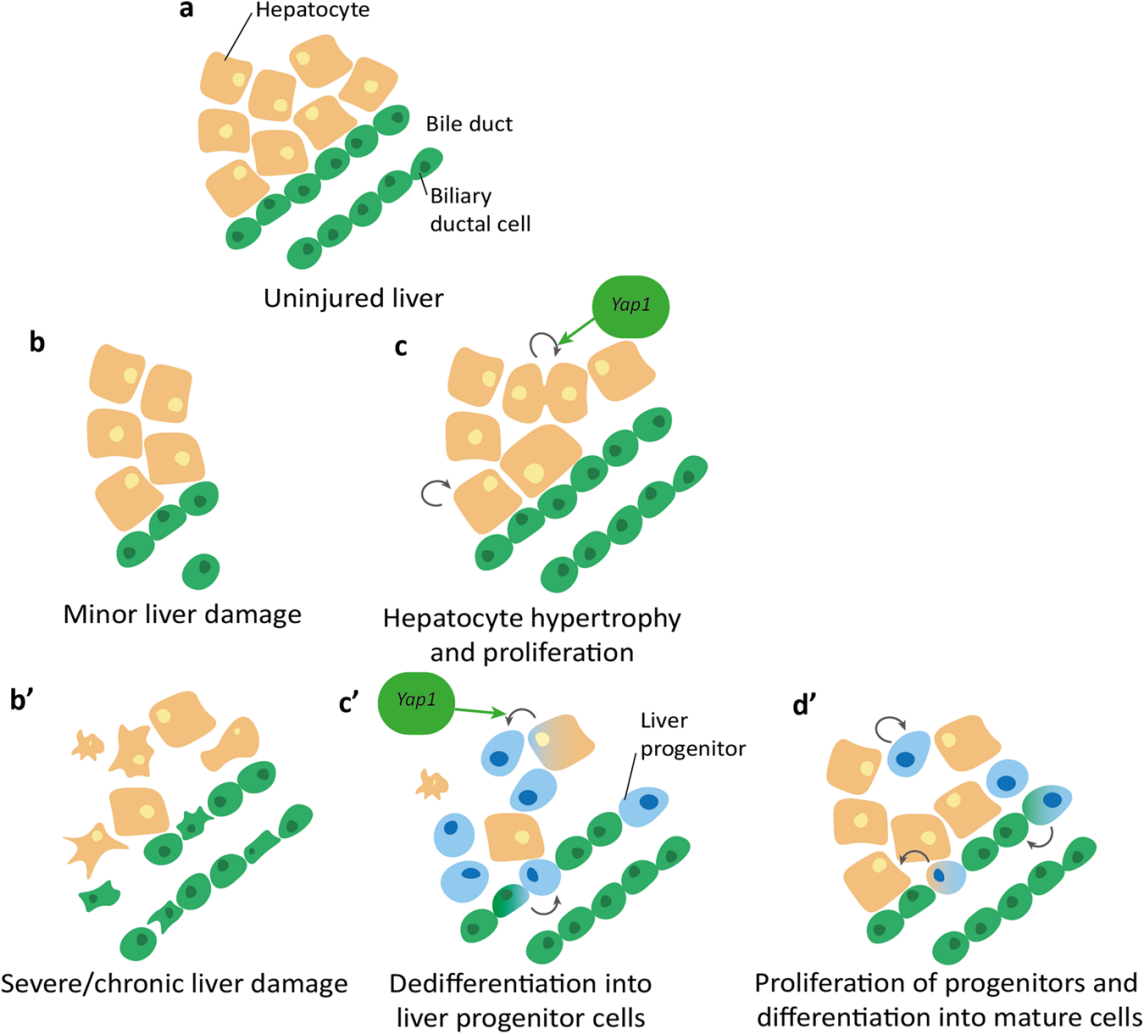
Overview of liver regeneration after minor (b, c) and severe (b’, c’, d’) injury. **a** Healthy (uninjured) zebrafish liver consists of multiple cell types hepatocytes (orange) and bile ducts comprising of biliary ductal cells (green). **b** Minor liver injury such as partial hepatectomy removes portions of the liver and the associated cells. **c** Liver recovery after minor liver damage involves hypertrophy and increased proliferation of remaining cells. *Yap1* promotes hepatocyte proliferation. **b’** Chronic or severe liver damage causes widespread cell death and necrosis. **c’** Remaining cells dedifferentiate into liver progenitor cells, promoted by *Yap1*. **d’** Progenitor cells proliferate then differentiate into mature hepatocytes and biliary ductal cells.

Despite limited mammalian regeneration of many organs, both mammals and zebrafish can regenerate their livers efficiently through the proliferation of differentiated hepatocytes, regaining liver function through epimorphic regrowth and compensatory enlargement of liver lobes^291,292^ (Fig. 7). However, this capacity of hepatocytes to repopulate the liver in humans can be overwhelmed by chronic or severe injury, resulting in liver failure that is only treatable by liver transplantation. There are many functional, cellular, and structural similarities between mammalian and zebrafish liver, both of which can regenerate their liver after more chronic insults,^292^ making the zebrafish a useful model to study the development and regeneration of the liver. However, limited research has been performed investigating the role of the Hippo pathway in zebrafish liver regeneration, although much work on this topic has been performed in the mouse. After experimental murine liver injury, YAP1 protein levels increase, with increased nuclear localisation in the liver and enhanced expression of downstream YAP1/TAZ target genes^220,293,294^. In a mouse model with *Yap* deletion in hepatocytes, bile duct ligation results in hepatic necrosis, reduced hepatocyte proliferation, and increased mortality^220,295^ compared to wild-type mice, suggesting a key role for Hippo signalling in mammalian liver regeneration.

One method posited to promote liver regeneration is the recapitulation of developmental processes to generate progenitor-like cells that repopulate the liver after hepatocyte loss. Supporting this, after severe liver injury biliary cells have been shown to transdifferentiate into hepatocytes via a dedifferentiated progenitor-like state to repopulate the liver^291,292^. Hippo signalling is implicated in multiple cell fate transitions during liver regeneration in the mouse^296,297^. This includes YAP signalling activation by the alteration of cholangiocytes’ epigenome and transcriptome to aid their restoration of normal hepatocyte and cholangiocyte number^296^. YAP also associates with factors such as Arid1a to promote the induction of liver progenitor-like cell-enriched genes^297^.

The Hippo pathway is linked to liver development in both mammalian and zebrafish livers, and likely regulates cell fate plasticity in this process. In mice, YAP1 overexpression causes hepatomegaly that is reversible upon cessation of YAP1 signalling, suggesting a function for YAP1 in regulation of cell proliferation and hepatocyte function^221,298^. Hepatomegaly is also observed in the zebrafish after Yap1 overexpression or *nf2a* disruption^299,300^, whilst conversely *yap1*^-/-^ fish have reduced liver size^147^. Other structural defects observed when disrupting upstream Hippo pathway components in the zebrafish include dilated bile ducts^299^, biliary dysgenesis^301^, and extrahepatic choledochal cysts^299^. Yap1 has also been linked to metabolism in the zebrafish liver, where it stimulates nucleotide biosynthesis to promote tissue growth through increasing glutamine synthetase and glucose transporter *glut1* expression^147,300,302^.

The Hippo pathway’s role in hepatocyte development is thought to be vital in its role in the liver as hepatocytes are the predominant cell type in the liver and are key to liver function^292^. Appropriate Hippo pathway function is essential in the maintenance of mature hepatocytes, with hepatocyte-specific *Nf2* loss in mice leading to hepatocyte dedifferentiation into highly renewable progenitors^303^, and overexpression causing a dysplastic hepatocyte morphology^221^. YAP1 is also associated with the formation of bile ducts in the developing mouse^304^, and with the function of the bile ducts (which promote immune cell recruitment and function) in the regenerating adult mouse liver^305^. Similarly, *stk3* and *sav1* zebrafish mutants (which both result in increased Yap1 activity) display altered hepatocyte morphology and polarity alongside biliary cell disruption^301^. Overall, these data suggest a conserved role for the Hippo pathway in structural liver, hepatocyte and biliary cell function between mammals and zebrafish. This implies that the Hippo pathway may also have a role in zebrafish liver regeneration, although this research is still in its infancy and will need further detailed investigation before conclusions can be drawn.

## Conclusion

The zebrafish is a powerful model system for the study of regeneration due to their rapid external development, relative low cost, transparent juvenile stages and robust reparative regeneration as well as the availability of a range of established genetic tools and other experimental procedures to study these. In this review, the role of the Hippo pathway in zebrafish regeneration is summarised, with the finding that Yap1/Taz signalling often enhances regeneration through the promotion of cell proliferation, progenitor cell dedifferentiation and maturation, EMT, and scar resolution, as well as linking to key developmental pathways. The phenotypes resulting from the disruption of Hippo pathway components is summarised in Table 2.

The positive effect of Yap1/Taz signalling on regeneration in the zebrafish, which appears to be latent in mammals, suggests some therapeutic potential in promoting YAP/TAZ signalling to enhance mammalian regeneration. However, this must be carefully investigated, as many of the processes associated with enhanced regeneration are linked to an increased risk of cancer, such as an elevated cell proliferation rate, cellular heterogeneity, and increased stemness^306^. In fact, dysregulation of the Hippo pathway and thereby pathological hyperactivation of YAP1/TAZ promotes carcinogenesis in most, if not all, types of solid tumours^102,307,308^. The zebrafish may therefore be vital in the elucidation of this association between cancer and regeneration, which could allow us to manipulate regenerative potential without impacting carcinogenesis or vice versa. One way to do this could be through the utilisation of zebrafish Hippo pathway-induced cancer models, which recapitulate human findings in that manipulation of Hippo signalling can trigger tumour formation^309–311^. However, the field of Hippo signalling in the zebrafish is still relatively new, and so much work must be performed to bridge the gaps that are currently preventing its translation to the clinic, particularly the study of the molecular and cellular drivers of the Hippo pathway’s effects on both regeneration and development (Box 1).

Box 1: Outstanding questions

1. What are the interactions and feedback between Hippo signalling and the immune system in regeneration that result in the current contradictory observations described? Does the Hippo pathway function differently in distinct immune processes, or are these apparent contradictions simply due to differences in experimental design?
2. Enhanced Yap1/Taz signalling is linked to increased risk of tumorigenesis. Is there a way to activate Yap1/Taz signalling spatiotemporally and precisely in specific cell types to promote regeneration without increasing cancer risk?
3. Can these findings be translated to humans? If so, can they be manipulated for therapeutic purposes, such as triggering regeneration in non-regenerating organs (e.g. heart and CNS) or promoting regeneration after chronic injury (e.g. liver)?
4. Zebrafish are an attractive model to study Hippo pathway dynamics in vivo, as it is amenable to live imaging with genetically encoded biosensors and tagged proteins. What are the dynamics of the Hippo pathway components in different cell types in vivo during development and regeneration in vertebrae?
